# Supplementary material for: Asynchronous magnetic resonance elastography: Shear wave speed reconstruction using noise correlation of incoherent waves
Source: Magn Reson Med. 2022 Oct 27;89(3):990–1001. doi: 10.1002/mrm.29502 (PMC9792433; doi:10.1002/mrm.29502)
Supplement: Supplementary file 1 — DATA S1 MATLAB code used to generate the numerical simulations of Figure 1 [file MRM-89-990-s002.zip › k-Wave/helpfiles/example_ewp_shear_wave_snells_law.html]

Shear Waves And Critical Angle Reflection Example (k-Wave)


# Shear Waves And Critical Angle Reflection Example

This example illustrates Snell's law for elastic media using a weakly focused ultrasound transducer incident on a soft-tissue / bone interface. It builds on the Explosive Source In A Layered Medium and Snell's Law And Critical Angle Reflection examples.

- Open the file in the MATLAB Editor
- Run the file in MATLAB

## Contents

- Elastic media and Snell's law
- Heating of bones by an angled transducer

## Elastic media and Snell's law

When a compressional wave in a fluid medium is incident upon a solid body, both compressional and shear waves are generated. For a plane wave source incident on a flat fluid-solid interface, the angle of the transmitted waves can be predicted based on the sound speeds in the two medium, where:

For an isotropic solid, there are two different sound speeds for the compressional and shear waves, with the compressional sound speed always being higher. This means the compressional and shear waves generated in the solid will travel at different angles.

When the incidence angle exceeds the critical angle

no wave is transmitted into the medium. As there are two sound speeds in the solid medium, there are two critical angles: one for the generation of compressional waves, and one for the generation of shear waves.

## Heating of bones by an angled transducer

Considering the case of bones and soft-tissue, the compressional sounds speeds are ~3000 m/s and ~1540 m/s, respectively. This gives a critical angle of ~30 degrees. Thus, if an ultrasound wave transmitted through soft tissue is incident on bone at an angle greater than 30 degrees, no compressional waves will be transmitted into the bone. However, the shear sound speed in bone is ~1400 m/s. This is close to the compressional sound speed in soft tissue, meaning shear waves will still be transmitted.

In this example, a weakly focused ultrasound transducer is incident on a soft-tissue / bone interface as illustrated in the figure below. The angle of the transducer is ~56 degrees, which is beyond the critical angle for the transmission of compressional waves into bone.

The simulation is run twice, first using the fluid code `kspaceFirstOrder2D`, and then using the elastic code `pstdElastic2D`. A visualisation of the square of the particle velocity magnitude (which is proportional to heating) is given in the figure below. The fluid code does not consider the propagation of shear waves, thus the incident wave is completely reflected from the interface. Conversely, using the elastic code illustrates a significant shear component inside the bone. The vastly different profiles illustrate the importance of considering shear waves for ultrasound simulations involving bones.
